# Targeting AKT as a promising strategy for SOX2-positive, chemoresistant osteosarcoma

**DOI:** 10.1038/s41413-024-00395-9

**Published:** 2025-02-24

**Authors:** Yujie Liu, Li Kang, Jing Luo, Minglei Yang, Da Wang, Juelan Ye, Xinghai Yang, Wei Wan, Jiemin Wong, Jianru Xiao

**Affiliations:** 1 Department of Orthopedic Oncology and Spine Tumor Center, Changzheng Hospital, Navy Medical University, Shanghai, 200001 China; 2 Naval Medical Center of PLA, Naval Medical University, Shanghai, China; 3 Shanghai Key Laboratory of Regulatory Biology, Institute of Biomedical Sciences and School of Life Sciences, East China Normal University, Shanghai, China; 4 Wuxi School of Medicine, Jiangnan University, Shanghai, China

**Keywords:** Bone cancer, Bone cancer

## Abstract

Osteosarcoma (OS) is the most prevalent type of primary malignant bone cancer and currently lacks effective targeted treatments. Increasing evidence indicates that SOX2 overexpression is a primary driver of OS. By screening a small-molecule kinase inhibitor library, we identified AKT as a kinase essential for robust SOX2 expression in OS cells. AKT was found to be frequently overexpressed in OS and positively correlated with SOX2 protein levels. We demonstrated that AKT has no effect on SOX2 transcription but promotes SOX2 protein stability. Mechanistically, AKT binds to and phosphorylates SOX2 at T116, preventing SOX2 ubiquitination and proteasome-dependent degradation by ubiquitin E3 ligases UBR5 and STUB1. Moreover, we found that AKT-SOX2 axis is a significant modulator of cancer stemness and chemoresistance and that the combination of AKT inhibitor MK2206 and cisplatin resulted in a synergistic and potent inhibition of OS tumor growth in the PDX model. In conclusion, we identified a critical role for AKT in promoting SOX2 overexpression, tumor stemness, and chemoresistance in OS, and provided evidence that targeting AKT combined with chemotherapy may hold promise for treating refractory OS.

**Figure:**
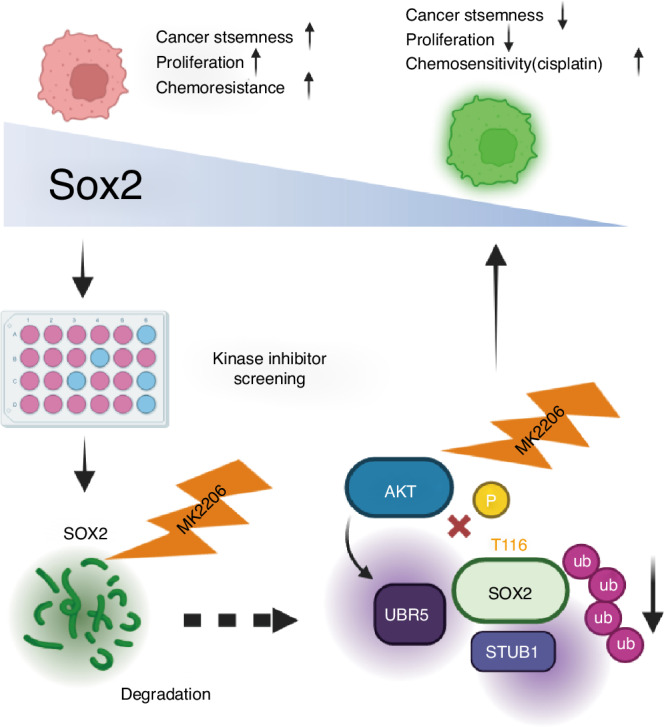
Working model showing that AKT stabilizes SOX2 by phosphorylating T116 site. Phosphorylation by AKT restraints the binding and ubiquitinoylation of SOX2 by the UBR5 and STUB1, thus promoting SOX2 stability and tumorigenic activity. Targeting AKT by MK2206 inhibits T116 phosphorylation and promotes SOX2 ubiquitination pathway, which impairs SOX2 tumorigenic activity. A combined treatment with chemo reagent and AKT inhibitor could achieve better therapeutic effect for SOX2-positive OS.

Working model showing that AKT stabilizes SOX2 by phosphorylating T116 site. Phosphorylation by AKT restraints the binding and ubiquitinoylation of SOX2 by the UBR5 and STUB1, thus promoting SOX2 stability and tumorigenic activity. Targeting AKT by MK2206 inhibits T116 phosphorylation and promotes SOX2 ubiquitination pathway, which impairs SOX2 tumorigenic activity. A combined treatment with chemo reagent and AKT inhibitor could achieve better therapeutic effect for SOX2-positive OS.

## Introduction

Osteosarcoma (OS) is an exceptionally aggressive malignant bone tumor that predominantly affects children and adolescents.^1,2^ OS has a high tendency for recurrence and early metastasis, particularly to the lung parenchyma.^3^ Standard OS treatment involves extensive surgical resection and chemotherapy, while radiation therapy is advised for unresectable tumors. Unfortunately, OS often develops resistance to conventional chemotherapies, leading to tumor recurrence and patient outcomes that have not improved over the past thirty years.^4–8^ In addition, several large clinical trials for intensified OS treatment in recent decades have failed to achieve a better cure rate.^9,10^ Therefore, novel therapeutic strategies are urgently required to overcome chemoresistance and enhance survival rates for OS patients.

Numerous studies have demonstrated that SOX2 overexpression contributes to cancer pathogenesis and progression, including cancer cell proliferation, metastasis, tumor initiation, and cancer stem cell function in various tumors, such as lung and esophageal squamous cell carcinomas. In osteosarcomas, high SOX2 expression has been associated with more aggressiveness, increased metastasis, chemotherapy resistance and poorer prognosis.^10–13^ Targeting SOX2 may eradicate the cancer stem cell population that is responsible for tumor recurrence after initial treatment. However, directly targeting SOX2 is technically difficult due to its nature as an “undruggable” transcription factor. Therefore, identifying and characterizing the molecular mechanisms leading to aberrant SOX2 overexpression in cancers could provide a basis for developing novel therapeutic approaches for various cancers driven by this abnormal overexpression.

SOX2 protein levels are widely regulated by post-transcriptional mechanisms. The AKT pathway is among the most frequently deregulated signaling pathways in human cancers. Activation of the AKT pathway contributes to tumorigenesis and metastasis by promoting cancer cell survival, proliferation, migration, and differentiation.^14^ Our previous research has shown that AKT plays a central role in stabilizing SOX2 proteins in mouse embryonic stem cells and esophageal cancer.^15,16^ Thus, it is worth exploring whether AKT regulates SOX2 stability and promotes cancer cell stemness and chemoresistance in OS.

In this study, we identified AKT as a kinase that has a critical role in promoting SOX2 protein stability and overexpression in OS. AKT is essential for stabilizing SOX2 by protecting it from proteasome-dependent degradation. We identified UBR5 and STUB1 as the main E3 ligases targeting SOX2 for proteasome-dependent degradation. We provide evidence that the AKT-SOX2 axis is crucial for cancer cell stemness and chemoresistance in OS. Finally, we showed in PDX models that a combined therapy strategy that targets AKT in conjunction with chemotherapy has a better therapeutic effect for OS.

## Results

### SOX2 is markedly upregulated in osteosarcoma and strongly correlates with patient prognosis

**Fig. 1:**
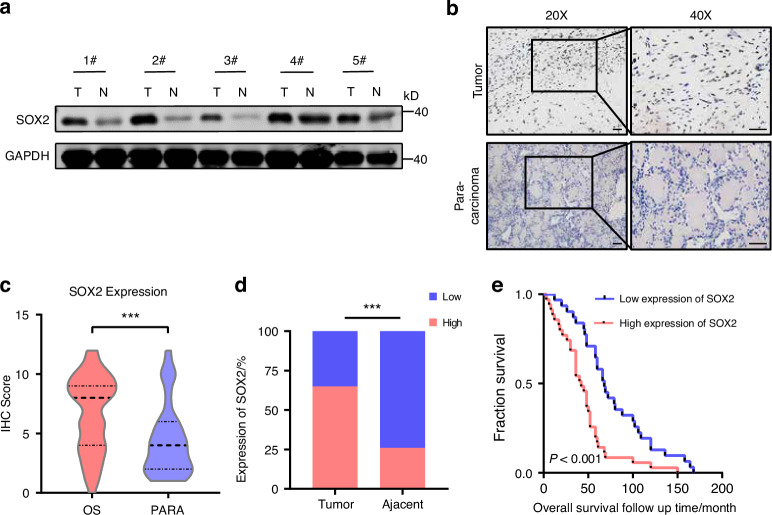
SOX2 expression is upregulated in OS tissues and related with poor prognosis. **a** Western blot images showing SOX2 highly expression in OS tissues. Coomassie brilliant blue is a control for protein loading. 3 representative blots are shown from five independent paired OS tissues. **b** Representative immunohistochemical images of SOX2 expression in osteosarcoma and para-osteosarcoma tissues. Scale bar, 50 μm (magnification, ×400). Statistical analysis for high expression rate (**c**) and IHC score (**d**) of SOX2 expression level in osteosarcoma and para-osteosarcoma tissues. **e** Kaplan–Meier plot of overall survival time analysis in patients with low and high expression of SOX2 in the Orthopedic Oncology Database cohort of Shanghai Changzheng Hospital. *P*-value was estimated using a log-rank test. ***P < 0.001; *P*-value was measured by *t* test

Several lines of evidence have showed that SOX2 overexpression occurs in various cancer. As shown in Fig. 1a, b, western blotting (WB) and immunohistochemical staining (IHC) results showed higher SOX2 expression in tumor tissues versus paracancerous tissues from OS patients in Orthopedic Oncology Department of Shanghai Changzheng Hospital. In addition, we found that the average IHC score of SOX2 was significantly higher in OS tumor tissues versus paracancerous (PARA) tissues (Fig. 1c). Statistical analysis showed that high expression rate for SOX2 is higher in tumor tissues versus paracancer tissues (Fig. 1d).

Furthermore, patients with OS were divided into SOX2-high and SOX2-low groups according to Fig. 1d. Notably, higher SOX2 expression in OS patients was related to poor overall survival (Fig. 1e), and Kaplan–Meier survival curves for sarcoma patients profiled in the GEPIA database also support this finding (Fig. S1A). Analysis of the detailed clinicopathological features of OS patients (Fig. S1B) showed that lung metastasis status, alkaline phosphatase (ALP) levels, and chemotherapy sensitivity differed based on SOX2 expression level. These results demonstrate that SOX2 is an adverse prognostic factor in human OS. Together, our data identified SOX2 as a potential crucial oncoprotein in OS.

### SOX2 promotes OS proliferation, migration and stemness in vitro

**Fig. 2:**
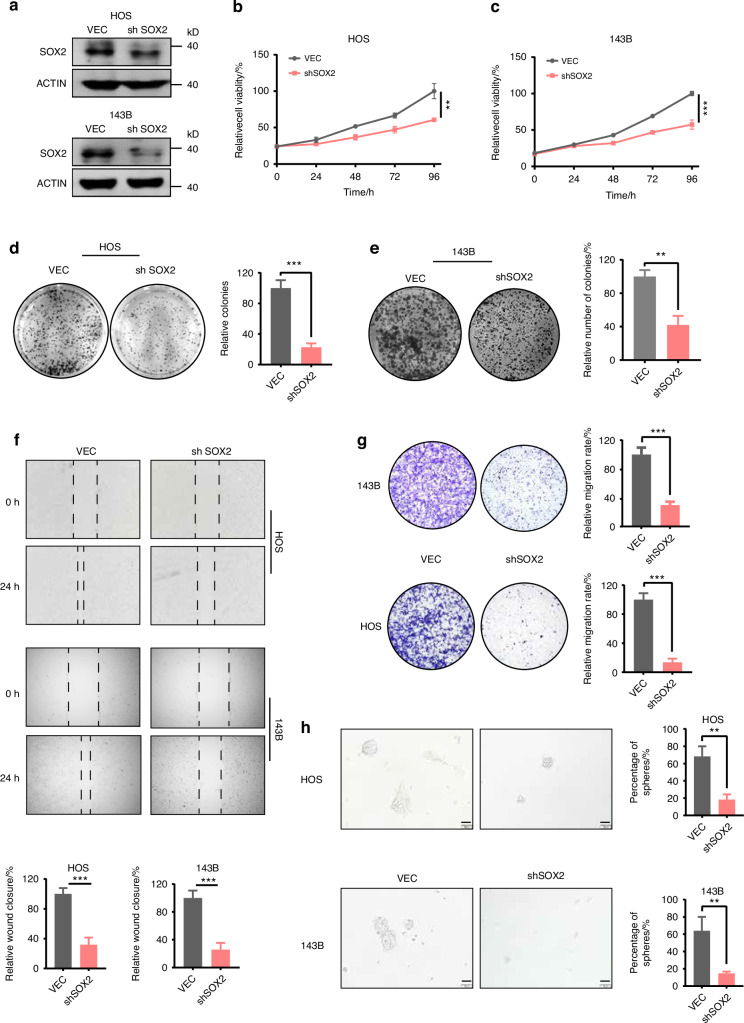
SOX2 promotes proliferation, migration and sarcomashpere ability of osteosarcoma cells in vitro. **a** Western blotting to detect the SOX2 knockdown efficiency. Cell proliferation ability for 143B and HOS cells after SOX2 knockdown. CCK8 assay (**b**, **c**), colony formation (**d**, **e**). Each value represents mean ± SD (*n* = 3). OS cells migration ability detection between for 143B and HOS cells after SOX2 knockdown. Wound healing images (**f**), and transwell migration (**g**). Each value represents mean ± SD (*n* = 3). **h** Sarcomasphere formation in control and SOX2-KD 143B and HOS cells. Each value represents mean ± SD (*n* = 3); *P < 0.05, **P < 0.01, ***P < 0.001; *P*-value was measured by *t* test

To determine the function of SOX2 in osteosarcoma, we validated the phenomenon in multiple OS cell lines in vitro using shRNA-mediated SOX2 knockdown (KD). As shown in Fig. 2a, shRNA against SOX2 substantially reduced its expression in OS cells. SOX2 silencing significantly decreased cell viability and proliferation in OS cells (Fig. 2b–e). Furthermore, we found that the migration ability was severely impaired in SOX2 KD cells compared to control cells (VEC) by wound healing assay (Fig. 2f) and transwell assay (Fig. 2g). Together, these data support that SOX2 maintains the ability of OS cell proliferation and migration.

Tumor stemness is closely linked to tumorigenesis and recurrence. The sphere-forming assay is a commonly used method in the cancer stem cell (CSC) field to enrich for cells exhibiting stem-like properties, especially when specific CSC biomarkers are undefined, such as in osteosarcoma.^17^ Therefore, we examined the presence of CSCs in a panel of osteosarcoma cell lines using the sphere-forming assay, which allows cancer stem cells to form tumor spheres while the majority of cancer cells do not survive. Of the four osteosarcoma cell lines tested, 143B and HOS, which exhibit high SOX2 expression, were able to efficiently form tumor spheres using a standard protocol. Initially, we assessed the impact of SOX2 knockdown on tumor sphere formation and observed that SOX2 knockdown in 143B and HOS cells significantly reduced tumor sphere formation (Fig. 2h). We then established SOX2-overexpression U2OS and MG63 cells which express relative low SOX2. The tumor sphere assay indicated that SOX2 overexpression not only increased the number of tumor spheres but also markedly enlarged their sizes (Fig. S2A-B). This finding highlights that SOX2 enhances tumor sphere formation and, consequently, cancer stem cell activity.

### Identification of AKT as a kinase required for robust SOX2 expression in OS cells

**Fig. 3:**
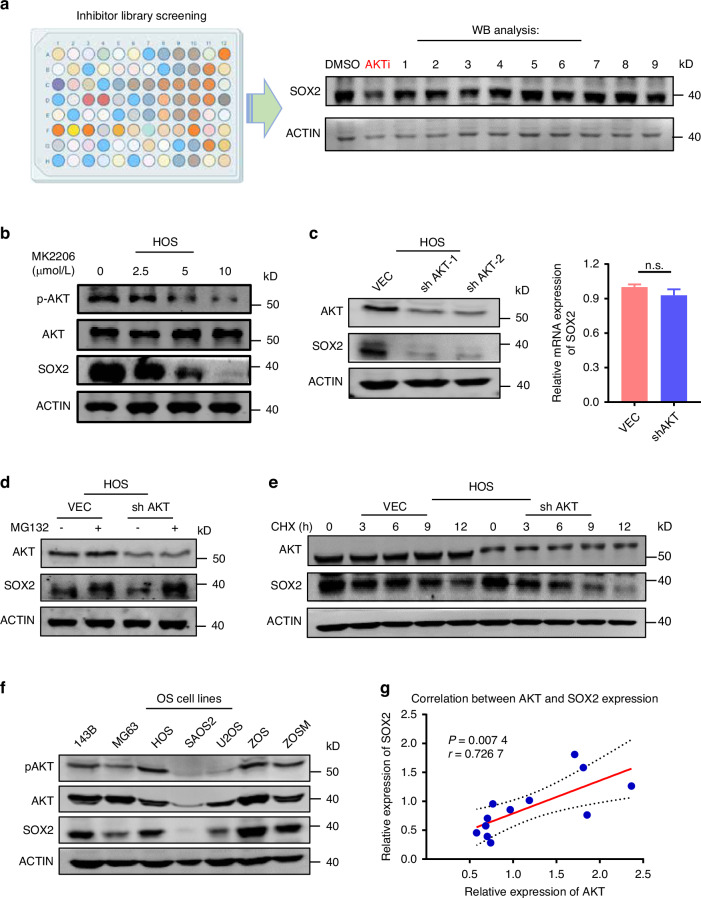
Identification of AKT as the potential positive regulator for SOX2 stability in OS. **a** Identification and characterization of AKT inhibitor as compound down-regulating SOX2 stability. The library contained 80 inhibitors for 30 major kinases. HOS cells in a format of 24-well plates were treated with each inhibitor at 10 μmol/L for 24 h before harvested for WB analysis. **b** WB analysis showing dose-dependent downregulation of SOX2 in HOS cells by AKT inhibitor. HOS cells were treated with MK2206 as indicated for 24 h and then subjected to WB analysis. Inhibition of AKT was confirmed by WB analysis using; actin is a control for protein loading. Representative blots are shown from 3 independent experiments. **c** AKT-knockdown cells were constructed with lentiviral shRNAs against AKT and detected for WB analysis and quantitative RT-PCR analysis which showed that protein level of SOX2 was downregulated and there was no significant downregulation of mRNA in HOS knockdown cells. Each value represents mean ± SD (*n* = 3). **d** Western blotting analysis showing MG132 blocked SOX2 downregulation after AKT-knocdown. MG132 was added at 10 μg/mL for 8 h before cell harvesting for WB analysis. **e** Control and AKT knockdown cells were treated with 100 μg/mL cycloheximide (CHX) for indicated time and harvested for western blotting analysis. Representative blots are shown from 3 independent experiments. **f** Western blotting analysis showing the levels of SOX2, AKT1 and active S473-phosphorylated AKT1 (pAKT1) in various osteosarcoma cell lines. **g** Correlation between AKT and SOX2 protein levels by protein profiling sequencing from the Orthopaedic Oncology Database of Shanghai Changzheng Hospital (12pairs). ***P < 0.001, *P* > 0.05, no significant (ns) correlation; *P*-value was measured by *t* test

Targeting SOX2 has been considered as a potential therapeutic strategy.^18–21^ Nevertheless, it is hard to target SOX2 directly due to its undruggable nature as a transcription factor. While phosphorylation by various kinases has been demonstrated to stabilize SOX2 in multiple cancers, it remains unclear whether the aberrant overexpression of SOX2 in osteosarcoma (OS) is partially attributable to enhanced stability through phosphorylation. To screen and identify the kinase(s) that contribute to SOX2 overexpression in OS cells, we treated SOX2-positive 143B cells with a library of kinase inhibitors that targeting 80 major cellular kinases. The AKT inhibitor emerged as the most effective agent for downregulating SOX2 in our screening (Fig. 3a, Fig. S4A). To validate the inhibitory effect of the AKT inhibitor on SOX2 protein levels, we treated SOX2-positive osteosarcoma (OS) cell lines, specifically HOS, with the AKT inhibitor MK2206 at varying concentrations for 24 h. We found that MK2206 down-regulated SOX2 protein levels with a dose-dependent manner (Fig. 3b).

To rule out potential off-target effects of the AKT inhibitor, we conducted additional experiments by employing two distinct AKT-specific short hairpin RNAs (shRNAs) to achieve AKT knockdown in HOS cells. We then assessed both the protein and mRNA expression levels of SOX2. We found that AKT knockdown dramatically reduced the SOX2 protein but not mRNA expression levels (Fig. 3c). Furthermore, suppression of SOX2 by knocking down AKT can be effectively blocked by addition of the proteasome inhibitor MG132 (Fig. 3d), indicating that inhibition of AKT promoted down-regulated SOX2 proteins through the proteasome degradation pathway. Consistent with this idea, the protein stability assay revealed that AKT knockdown dramatically reduced the half-life of the SOX2 protein in HOS cells from approximately 9 h to less than 6 h (Fig. 3e). Similar assays were performed with 143B cells and we observed the consistent results (Fig. S4B-E).

To evaluate the relevance of SOX2 and AKT1 in OS, we collected a panel of seven OS cell lines and examined the correlation of SOX2, AKT1 and active form of S473-phosphorylated AKT1 by western blotting (Fig. 3f). SOX2 protein level showed a positive correlation with protein level of AKT1 or S473-phosphorylated AKT1 in OS cell lines. Furthermore, we also analyzed the correlation of SOX2 and AKT1 proteins in 12 human osteosarcoma protein profiling sequencing from the Orthopedic Oncology Database of Shanghai Changzheng Hospital. Consistently, the SOX2 protein levels were positive correlated with AKT (Fig. 3g). These data collectively suggest that SOX2 expression is positively correlated with AKT1 in cell lines and OS tumors.

**Fig. 4:**
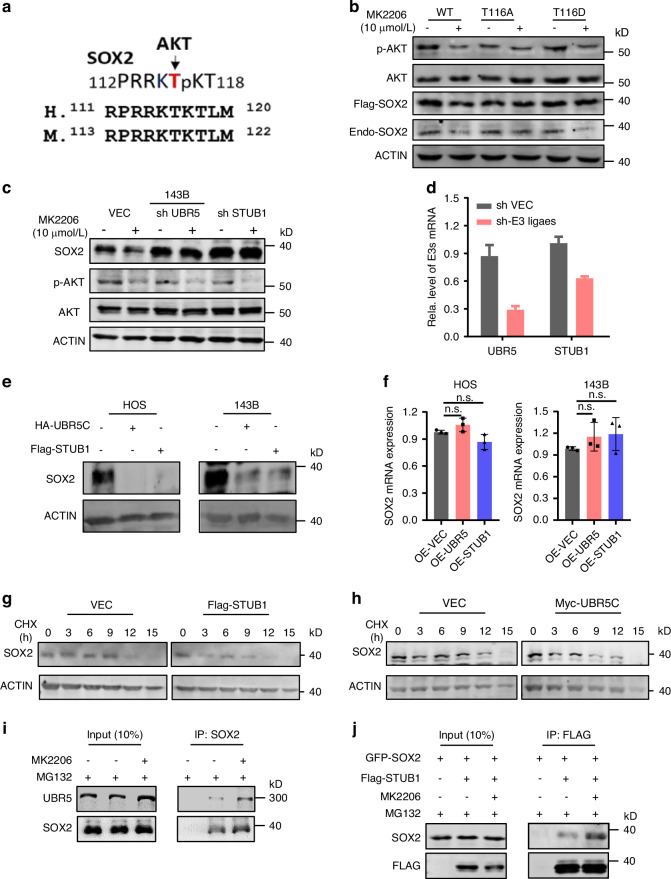
AKT phosphorylates SOX2 and inhibits the interaction with and ubiquitination by E3 ligases to stabilize SOX2 stability. **a** The diagram showing that SOX2 phosphorylation site at T116. **b** The phosphorylation-mimic T116D mutant is resistant to UBR5C-induced SOX2 ubiquitination. The wild-type or phosphorylation-mimic mutant SOX2 (T116A or T116D) was transfected into HOS cells and the stable cells were established by lentiviral infection of the corresponding constructs. The cells were treated with or without MK2206 (10 μmol/L) for 24 h and harvested for western blotting analysis as indicated. **c** Different ubiquitin ligases (E3s) were knocked down instantaneously in HOS cells and expressed continuously for 3 days. Then each group was treated with MK2206 (10 μmol/L) for 24 h, and the level of SOX2 protein was detected by western blot. **d** Quantitative RT-PCR analysis to detect the mRNA level of SOX2 after E3s knockdown. **e** Two different E3s were overexpressed in OS cell lines to detect the protein level of SOX2 expression using WB analysis. **f** Quantitative RT-PCR analysis to detect the mRNA level of SOX2 after E3s overexpression. Western blot images: Representative blots are shown from 3 independent experiments. Each value represents mean ± SD (*n* = 3). **P < 0.01, ***P < 0.001; *P*-value was measured by *t* test. 143B cells were transfected with or without Flag-STUB1(**g**) or Myc-UBR5C (**h**) and cultured for two days. 143B cells were then treated with CHX (100 μg/mL) at various times as indicated and subjected to WB analysis. **i** 143B cells were treated with MG132 to block SOX2 degradation and with or without 2 μmol/L MK2206 for 24 h. The cellular extracts were prepared and subjected to IP with or without SOX2 antibody. **j** 143B cells were transfected with GFP-SOX2 alone or together with Flag-STUB1 for 24 h and treated with or without 2 μmol/L MK2206 for another 24 h. Then 10 μg/mL MG132 was added for another 8 h before cells were harvested for IP-WB analysis

### AKT phosphorylates and protects SOX2 from E3 ligase-mediated ubiquitination and degradation in OS

A previous study had reported that AKT phosphorylates SOX2 at T116 and blocks the interaction of SOX2 with UBR5, which protects SOX2 from ubiquitin-dependent protein degradation by UBR5 in human esophageal cancer (Fig. 4a). To determine whether AKT-regulated SOX2 stability is dependent on phosphorylation at T116 in osteosarcoma, we established stable 143B cell lines expressing FLAG-tagged SOX2 or its mutants (T116A and T116D) and treated them with MK2206. Western blot analysis showed that MK2206 treatment effectively reduced the levels of wild-type SOX2, whereas it had no impact on the levels of the T116A and T116D mutants (Fig. 4b). These results indicate that AKT stabilizes SOX2 by phosphorylating it at T116. In view of the previous study, we further evidenced if AKT stabilized SOX2 by antagonizing SOX2 degradation induced by UBR5 or other E3 ligase(s) in OS cells. To this end, not only UBR5 but also CHIP, another SOX2’s E3 ligase,^22^ had been knocked down by corresponding specific shRNA in 143B cells and treated the cells with or without MK2206 (Fig. 4c). The effect of each shRNA was verified by RT-PCR analysis (Fig. 4d). We found that suppression of UBR5 and CHIP could block MK2206-induced degradation of SOX2, suggesting UBR5 and CHIP both play crucial roles in AKT-mediated SOX2 stability in OS cells.

We next examined whether the UBR5 and CHIP regulate SOX2 ubiquitination and stability in OS. We found that ectopic overexpression of UBR5 and CHIP markedly downregulated the SOX2 proteins and had minimal effect on SOX2 mRNA levels (Fig. 4e, f). Furthermore, UBR5 and CHIP overexpression reduced the half-life of SOX2 proteins from approximately12 h to 3 h in 143B cells (Fig. 4g, h)

In our study, both UBR5 and CHIP were involved in AKT-induced SOX2 stability in OS cells. Therefore, we examined whether the interactions of SOX2 with UBR5 and CHIP could both be influenced by SOX2-T116 phosphorylation. We firstly analyzed the interaction between endogenous SOX2 and UBR5 using cellular extracts derived from 143B cells. Consistent with the results in esophageal cancer cells, MK2206 treatment significantly enhanced the interaction UBR5 and SOX2. Additionally, the binding of ectopically expressed CHIP and SOX2 could also be enhanced upon MK2206 treatment (Fig. 4i, j). In summary, we concluded that AKT protects SOX2 from degradation by blocking the interaction of SOX2 with UBR5 and CHIP in OS cells.

**Fig. 5:**
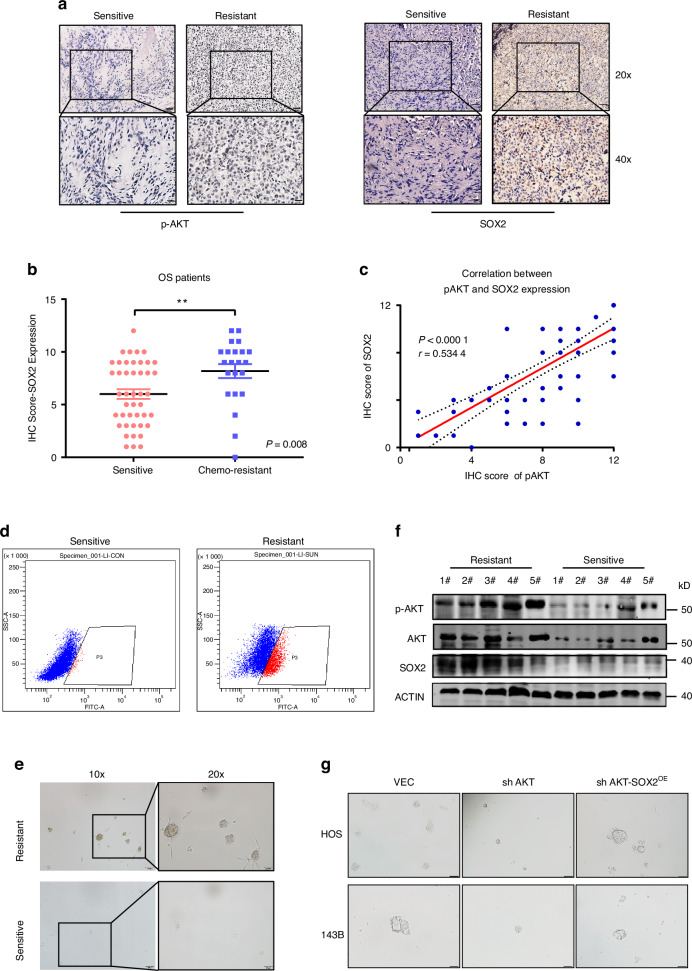
AKT-SOX2 axis shows crucially positive regulation in chemo-resistant OS. **a** Representative immunohistochemical images of SOX2 expression and p-AKT expression in chemo-resistant and sensitive OS tissues. SOX2 protein levels was positive correlated with p-AKT expression in OS tissues. **b** IHC score of SOX2 expression level in chemo-resistant and sensitive OS tissues. **c** Correlation between SOX2 and p-AKT expression by IHC score from the Orthopedic Oncology Database of Shanghai Changzheng Hospital. **d** FACS side scatter analysis of percentage of ALDH^+^ cells in primary OS cells derived from chemo-resistant or sensitive OS tissues. **e** Western blot images showed that p-AKT and SOX2 expression in chemo-resistant and sensitive OS tissues. **f** Sarcomasphere detection to evaluate the tumor stem cell formation ability for primary OS cells derived from chemo-resistant or sensitive OS tissues. **g** Sarcomasphere detection to evaluate the tumor stem cell formation ability for stable cell lines of VEC, sh AKT and sh AKT-SOX2^OE^. ***P < 0.001, *P* > 0.05, no significant (ns) correlation; *P*-value was measured by *t* test

### AKT-SOX2 axis is important for cancer stemness and chemosensitivity in OS

Osteosarcoma stem cells are thought to play pivotal roles in tumor recurrence, metastasis, and chemoresistance through mechanisms of self-renewal and differentiation. Given that CSCs may be more resistant to chemotherapy, it’s important to clarify the regulatory mechanism in OS. SOX2 has been implicated in promoting cancer stem cell function in various cancers and we have also proven it in the previous results. In previous results, we found that AKT could phosphorylate to protect SOX2 protein stability and knockdown AKT could inhibit the stemness ability in OS cells.

Moreover, we detected the expression of SOX2 and p-AKT in OS tissues to verify the relationship between AKT-SOX2 axis with chemoresistance. Figure 5a showed that high expression of SOX2 and p-AKT in chemoresistant OS tissues, which was the reverse in the sensitive group. In addition, IHC score for SOX2 (Fig. 5b) were significantly higher in OS chemoresistant tissues. Correspondingly, high SOX2 expression in tissues was accompanied by high expression of p-AKT (Fig. 5c). The connection between SOX2 and p-AKT was further examined by the IHC score in the Changzheng Hospital Orthopedic Oncology database. As shown in Fig. 5c, the R-value at 0.534 4 reveals a positive correlation between the two proteins in chemo-resistant patients with OS.

CSCs were FACS sorted from OS patients’ tumors. ALDH^+^ cells were enriched and differences in CSCs levels between chemo-resistant tissues and sensitive tissues were shown in Fig. 5d. Similar observations were made in vitro (Fig. 5e). Furthermore, SOX2 and p-AKT expression were upregulated (Fig. 5f) in chemo-resistant tissues compared with sensitive tissues. To determine whether SOX2 contributes to the AKT-abrogated cellular phenotype, we assessed oncosphere formation ability in AKT-KD cells when SOX2 was overexpressed. Importantly, we demonstrated that the stemness effect was successfully reversed in AKT-KD-SOX2-overexpression cells (Fig. 5g).

**Fig. 6:**
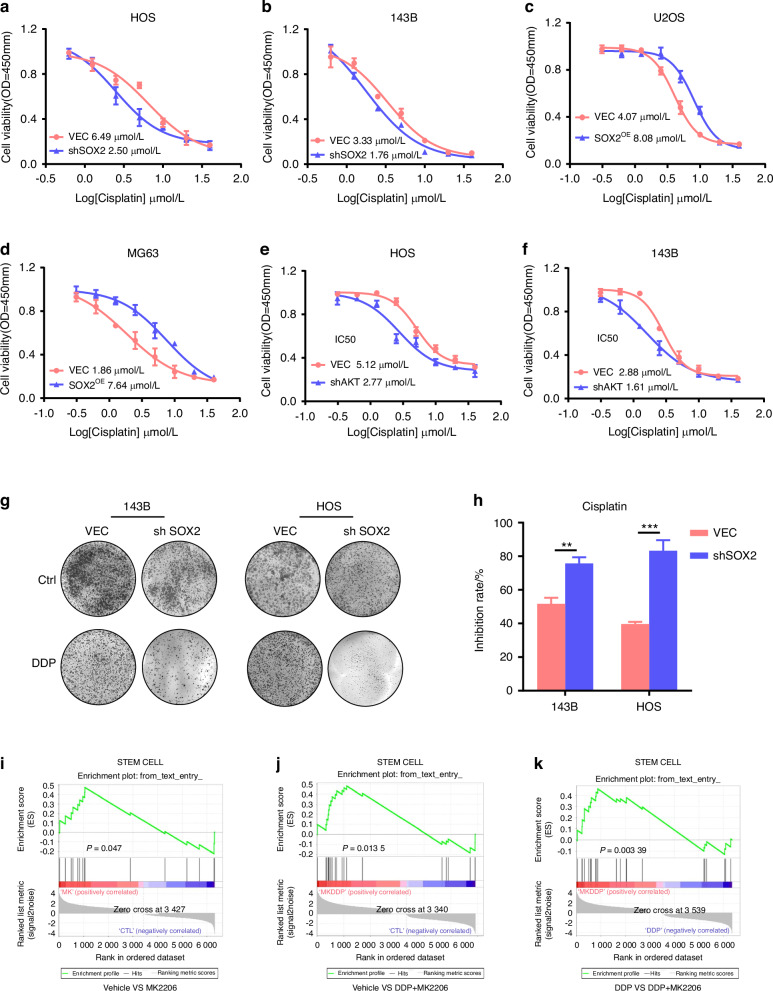
AKT-SOX2 deficiency enhances sensitivity of OS cells to cisplatin. **a**, **b** Cell viability for ctrl and SOX2 knockdown cells with cisplatin treatment. **a** HOS cell line; **b** 143B cell line; Relative intensity measured at OD 450 nm. **c**, **d** Cell viability for ctrl and SOX2 overexpression cells with cisplatin treatment. **c** U2OS cell line; **d** MG63 cell line; Relative intensity measured at OD 450 nm. **e, f** Cell viability for ctrl and AKT knockdown cells with cisplatin treatment. **e** HOS cell line; **f** 143B cell line; Relative intensity measured at OD 450 nm. **g** Colony formation assay to detect the antitumor effect of cisplatin in ctrl and SOX2 knockdown cells. **h** Inhibition rate comparison for cisplatin in ctrl and SOX2 knockdown cells. Each value represents mean ± SD (*n* = 3). **P < 0.01, ***P < 0.001; *P*-value was measured by *t* test. **i**–**k** GSEA analysis of stem cell signaling for DDP or MK2206 treatment

To investigate the role of SOX2 in chemoresistance, we employed stable shRNA-mediated knockdown and transient overexpression of SOX2. Cisplatin (DDP), a standard chemotherapeutic agent for osteosarcoma (OS), was used to assess its impact on cell survival. Cell viability in response to DDP was evaluated using CCK8 assays in both control (shCtrl) and SOX2 knockdown (shSOX2) OS cell lines. Compared to shSOX2 cells, which exhibited increased sensitivity to cisplatin, SOX2-overexpressing OS cells demonstrated at least a two-fold increase in the DDP IC50 value (Fig. 6a–d). Furthermore, shSOX2 OS cells became more sensitive to DDP (Fig. 6g, h), suggesting the SOX2 pathway is a common signature of Pt-resistant cells. Then, we constructed AKT-knockdown cell lines to detect the AKT function in OS cells. Similarly, AKT KD in OS cells decreased cell viability (Fig. S2C, D), colony formation (Fig. S2E, F), migration ability (Fig. S2G, H), and sphere formation (Fig. 5g). Notably, high AKT expression is a poor prognostic factor in sarcoma patients (Fig. S3A). We observed that AKT knockdown (KD) significantly inhibited tumor growth and progression in a tibia tumor-bearing model (Fig. S3B, C). At the conclusion of the experiment, tumors were excised, and their weights were recorded (Fig. S3D), corroborating that AKT KD markedly impedes osteosarcoma (OS) tumor progression. Additionally, Western blot analysis revealed decreased SOX2 levels in xenograft tumors derived from shAKT-expressing OS cells relative to tumors from control cells (Fig. S3E). Moreover, AKT KD cells were more sensitive to DDP and decreased IC50 to DDP by nearly 2-fold (Fig. 6e, f).

Our data indicate that a chemoresistant cancer cell population, which acts as a reservoir for resistant tumors, exhibits characteristics typical of cancer stem cells (CSCs) and is marked by SOX2 expression. We show that the survival of these cells relies on an active AKT-SOX2 signaling pathway, which contributes to their resistance to cisplatin. These findings underscore the significance of the AKT-SOX2 axis in regulating cancer stemness and chemoresistance in osteosarcoma.

### MK2206 overcomes stemness and synergizes with cisplatin in OS treatment

**Fig. 7:**
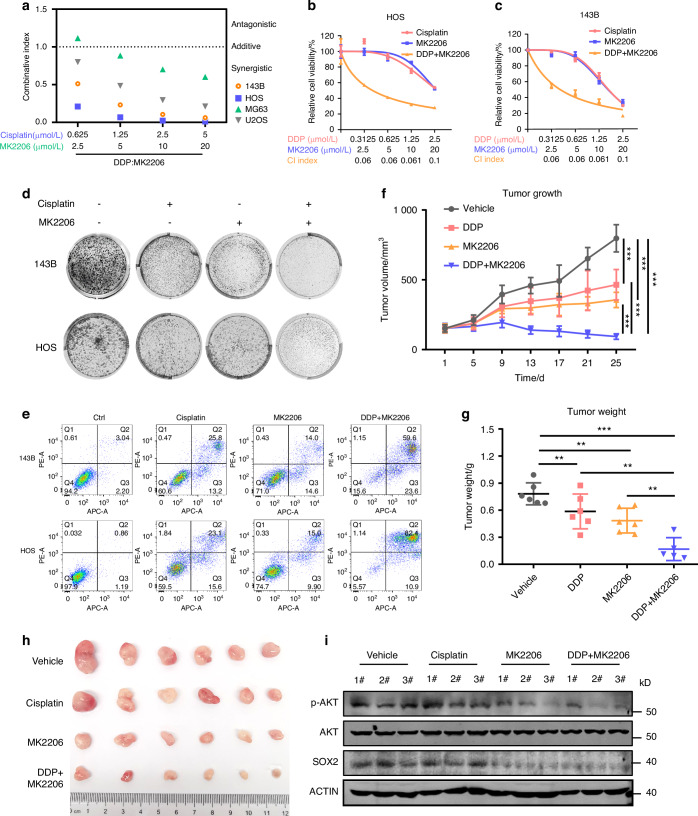
MK2206 overcomes the chemoresistance and synergizes with *cisplatin* in vitro and in vivo. **a** Relative combination rate for MK2206 and cisplatin combination therapy. Cell viability (**b, c**) and colony formation (**d**) of 143B and U2OS cells with or without MK2206 or cisplatin treatment. Relative intensity measured at OD 450 nm. Each value represents mean ± SD (*n* = 3). **e** Apoptotic rates induced by cisplatin, MK2206 or combination for 24 h in OS cells were detected by annexin V/PI double‐staining assay. **f** Statistical analysis of tumor volume of PDX tumors model with or without MK2206 or cisplatin combinative therapy. Each value represents mean ± SD (*n* = 6). **P < 0.01, ***P < 0.001; *P*-value was measured by a *two-way ANOVA analysis*. Comparison of therapeutic effects in four treatment groups after the euthanasia for PDX model. Statistical analysis of tumor weight (**g**), the image of xenograft tumors in four groups (**h**). Each value represents mean ± SD (*n* = 6). **P < 0.01, ***P < 0.001; *P*-value was measured by *t* test. **i** Western blot images showing p-AKT and SOX2 protein level with or without MK2206 and cisplatin combination therapy in PDX model. Representative blots are shown from 3 independent experiments

In the previous work, we demonstrated the important role of AKT in regulating the stability of SOX2 protein and the important role of the AKT-SOX2 axis in osteosarcoma stemness. Thus, we use MK2206 as a candidate drug to inhibit osteosarcoma cells. Western blots showed SOX2 expression and sphere formation capacity of osteosarcoma cells decreased after MK2206 addition. After cisplatin treatment, SOX2 expression slightly increased and sphere formation ability increased (Fig. S5A-B). Moreover, GSEA analysis demonstrated MK2206 inhibited the stemness pathway in OS cells (Fig. 6i–k). Therefore, we hypothesized combining MK2206 could reduce stemness by inhibiting SOX2, achieving combined treatment effects. As shown in Fig. S6A-C, MK2206 can significantly inhibit osteosarcoma cell proliferation and clonogenesis of osteosarcoma cells, and its IC_50_ in osteosarcoma cells ranges from 9.7-24.5 μmol/L. Furthermore, the combination experiment showed MK2206 combining cisplatin had good synergy with combination index. As shown in Fig. 7a–c, both drug combination coefficients were < 1. Figure 7d and S7A, B showed that MK2206 combined cisplatin significantly inhibited colony formation of osteosarcoma cells. Figure 7e and S7C, D showed MK2206 combining cisplatin significantly increased the apoptosis of osteosarcoma cells.

We also observed that the combined treatment of 10 mg/kg MK2206 and 2 mg/kg cisplatin was well tolerated by mice and more effective in suppressing tumor progression compared to either cisplatin or MK2206 administered alone. Both in vivo and cell experiments showed that MK2206 and cisplatin alone could slightly inhibit the growth of osteosarcoma PDX, and the inhibitory effect was very significant when combined (Fig. 7f–h). Figure S7E showed that the weight of mice with this treatment regimen did not decrease significantly. WB and IHC tests in tumor tissues (Figs. 7i and S7F) further confirmed that the protein expression of p-AKT and SOX2 decreased when MK2206 was added.

## Discussion

The underlying mechanism remains poorly understood and OS still lacks approved targeted therapies.^23^ Elucidation the molecular mechanism may help identify novel prognostic biomarkers and facilitate developing effective therapies against OS. Given that aberrant SOX2 expression has been strongly implicated as a critical oncogenic driver in osteosarcoma (OS),^24–28^ our objective was to identify and characterize the signaling pathways responsible for inducing SOX2 overexpression in OS. Screening a kinase inhibitor library led to the identification of AKT as a key kinase involved in SOX2 overexpression in OS cells. Mechanistic studies demonstrated that AKT enhances SOX2 stability by phosphorylating SOX2 at T116 and inhibiting its interaction with UBR5 and STUB1. Importantly, we present evidence that AKT-SOX2 axis exhibits a potential therapeutic feasibility against SOX2-positive OS tumors and AKT inhibitor combined with cisplatin show significant synergistic therapeutic effect in PDX model. Indeed, our findings have several implications.

SOX2 overexpression have been broadly observed in various cancers and treated as a critical diver in cancer occurrence and progression.^29–31^ In osteosarcoma, it has been reported that aberrant expression of SOX2 promotes tumor initiation and proliferation.^24,32^ In this study we found SOX2 is highly expressed in osteosarcoma tissues and correlates poor survival of OS patients (Fig. 1) and plays an essential role in tumor growth, metastases and chemoresistance (Figs. 2, 5 and 6), indicating that SOX2 is a central target for OS treatment. Subject to SOX2 as an undruggable transcriptional factor, we carried on a screening library with different kinase inhibitors and found AKT inhibitor can dramatically downregulate the levels of SOX2. Indeed, previous studies have shown that AKT can regulated SOX2 expression in non-small lung cancers^33^ and enhanced SOX2 protein levels in hippocampal neural progenitor cells.^34^ Besides, our previous study had reported that AKT phosphorylates SOX2 at T116 and blocks the interaction of SOX2 with UBR5, which protects SOX2 from ubiquitin-dependent protein degradation by UBR5 in human esophageal cancer.^16^ It worth noting that it exits not identical mechanism of AKT-mediated SOX2 stability in OS cells. In addition to UBR5, we found STUB1-induced SOX2 degradation can also be blocked by phosphorylation of SOX2 at T116. In the end, our study reveals AKT-mediated protein stabilization is a major determinant for SOX2 overexpression. Thus we develop a new insight that AKT can be as a potential target for SOX2-positive OS.

We found that elevated SOX2 expression positively correlates with drug resistance and poor survival of OS patients (Figs. 1, 5). Mounting evidence indicates that a small subset of cancer cells with stem cell-like properties, termed cancer stem cell (CSCs), drive tumorigenesis, metastasis, and recurrence.^35,36^ CSCs represent the sole fraction of the tumor cell population with the capacity to initiate tumor growth, continuously producing non-tumorigenic progeny that constitute the majority of the tumor. CSCs are believed to contribute to chemotherapy resistance in numerous tumors, making selective targeting of CSCs a promising strategy for achieving tumor eradication and potential cure.^37,38^ Osteosarcoma stem cells are believed to play pivotal roles in tumor recurrence, metastasis, and chemoresistance through mechanisms of self-renewal and differentiation. Targeting these stem cells could offer a novel therapeutic strategy for the treatment of osteosarcoma. SOX2 is a critical factor in preserving stemness and has been implicated in sustaining the undifferentiated tumorigenic state across various cancers.^39^ Several studies have demonstrated that this stem cell transcription factor is essential for maintaining cancer stem cells (CSCs) in sarcomas.^13,24,40^ Importantly, our immunohistochemical results showed higher expression of SOX2 and p-AKT in tumor samples from chemotherapy-resistant patients compared to chemotherapy-sensitive patients.

What’s more, AKT-SOX2 axis is strongly associated with maintaining stemness and enhancing chemotherapeutic resistance in OS. Both loss-of-function and gain-of-function experiments convinced us that the AKT-SOX2 axis is critical for the chemoresistance in OS cells. We found that the AKT-SOX2 axis was activated and marks a population exhibiting characteristics of cancer stem cells in chemo-resistant OS tumors. Besides, GSEA analysis demonstrated that cancer stem cell pathway was upregulated following cisplatin treatment, but was inhibited after adding the AKT inhibitor. Our current findings corroborate the link between AKT-SOX2 axis and cancer stemness and identified this axis as facilitating of drug resistance in OS. Knockdown of SOX2 sensitized OS cells to platinum, while SOX2 overexpression conferred platinum resistance in the cells. Therefore, the activation of AKT mediated by SOX2 likely plays a significant role in the development of resistance to DNA-damaging agents in osteosarcoma. The observation demonstrated that AKT-SOX2 is responsible for the sensitivity of cisplatin in OS cells and underscores the potential therapeutic benefit of targeting the AKT-SOX2 axis in the treatment of osteosarcoma.

Furthermore, we demonstrated that MK2206 as a AKT inhibitor can significantly inhibit the stemness of osteosarcoma cells, and combined cisplatin therapy has shown synergistic inhibition effect in vitro and in vivo. Cisplatin remains a first-line drug for OS chemotherapy. However, a major factors contributing to chemotherapy failure is the development of drug resistance,^41^ which affects prognosis and patients survival. Generally, most cancer cells are initially susceptible to platinum, but they commonly develop resistance over time. These tumors only respond to high doses of chemotherapy and acquire resistance rapidly, as evidenced by the low salvage rates of only 20% in patients with relapsing disease.^42^ Our results suggest the AKT-SOX2 axis as a potential key regulator of cancer cell populations that are prone to survive platinum exposure, enriched with antioxidant response mechanisms. These rare cancer cell subpopulations that are responsible for disease relapse after chemotherapy exhibit stem cell-like features and can be eradicated by inhibiting AKT.

We demonstrated that SOX2 overexpression leads to tumor chemoresistance in osteosarcoma tumors.^43^ Our immunohistochemical results revealed higher SOX2 expression in chemotherapy-resistant osteosarcoma patients compared to chemotherapy-sensitive patients, suggesting that combining AKT inhibitor with chemotherapy may benefit treatment. Previous studies have provided some evidence that increasing cisplatin sensitivity by schedule-dependent inhibition of AKT and Chk1.^44^ As expected, combined MK2206 and cisplatin significantly reduced tumor growth in the OS patient-derived xenograft (PDX) mouse model. Our study provides insights into how targeting the AKT pathway along with chemotherapy may improve antitumor activity in osteosarcoma. Several AKT inhibitors have been developed and are currently undergoing evaluation in clinical trials for cancer treatment. Our results warrant conducting clinical trials combining AKT inhibitors and chemotherapy to treat osteosarcoma patients, which could improve outcomes. In summary, the combination of AKT inhibition with chemotherapy demonstrate potential for improving osteosarcoma treatment. We recommend further exploring this combination approach through clinical trials to benefit osteosarcoma patients.

In this study, we identified AKT as a critical oncogenic kinase responsible for the aberrant overexpression of SOX2 in osteosarcoma (OS). This study identifies a promising therapeutic target and elucidates the crucial role of the AKT-SOX2 pathway in OS progression. Moreover, we found that AKT-SOX2 axis is a modulator of cancer stemness and chemoresistance and developed a combination strategy using an AKT inhibitor to decrease SOX2 protein level and enhance OS sensitivity to chemotherapeutic drugs. In summary, we have elucidated the crucial role of AKT in promoting SOX2 overexpression, enhancing tumor stemness, and conferring chemoresistance in osteosarcoma (OS). Our findings provide substantial evidence that targeting AKT in conjunction with chemotherapy holds potential for the treatment of refractory OS.

## Materials and methods

### Antibodies and inhibitors

The antibodies used in this study were: anti-SOX2 (Abcam, #92494), anti-AKT (Absci, #abs4582), anti-p-AKT (Proteintech, #66444-1-Ig), anti-β-Actin (HUABIO, #M1210-2), anti-FLAG (Sigma, #F1804), and anti-HA (Abmart, #M20003). The secondary antibodies included: Alexa Fluor 680 goat anti-rabbit IgG (Jackson ImmunoResearch, #111-625-144), Alexa Fluor 790 goat anti-mouse IgG (Jackson ImmunoResearch, #115-655-146), and Alexa Fluor 488 goat anti-mouse IgG (Jackson ImmunoResearch, #115-545-003). The AKT inhibitors MK-2206 (#HY-10512) and cisplatin (#HY-17394) were obtained from MCE.

### Plasmid construction

All plasmids were confirmed by DNA sequencing. Expression plasmids for SOX2 and STUB1 were constructed in the pCDH vector for lentivirus packaging (stable expression) as described. The UBR5C plasmid was generously provided by Professor Shimin Zhao at Fudan University. All mutant expression constructs were generated by site-directed mutagenesis and confirmed by DNA sequencing. The shRNA constructs targeting human SOX2, AKT, UBR5, and STUB1 were generated by cloning the corresponding shRNA coding sequences into the pLKO.1 vector. The sequences of the shRNAs are detailed in Table S1.

### Kinase inhibitor screening

Human osteosarcoma HOS cells (1 × 10^5^ cells per well) were seeded in 24-well plates, cultured in DMEM medium with 10% FBS, and treated with various compounds from Kinase Inhibitor Libraries (Enzo Life Sciences, Merck KGaA, Selleck Chemicals, Table S2) at a final concentration of 10 μmol/L for 24 h. The cells were then collected and subjected to Western blot analysis for SOX2. The screening was repeated three times, and a positive hit was defined as a compound that downregulated SOX2 in all three experiments.

### Cell transfection and stable cell line establishment

Human osteosarcoma HOS, U2OS, MG63, ZOS, SAOS-2, ZOSM, 143B cells, and Human Embryonic Kidney 293 T cells were cultured in Dulbecco’s Modified Eagle Medium (DMEM; Gibco) supplemented with 10% fetal bovine serum and 100 U/mL penicillin/streptomycin solution (Gibco). All cell lines were cultured at 37 °C in a humidified atmosphere containing 5% CO2. Transient transfection was carried out using LipoFiter (Hanbio) following the manufacturer’s instructions. For the generation of stable cell lines via lentiviral infection, a mixture of the three-plasmid system (psPAX2, pMD2.G, and transfer plasmid) was prepared and transfected into 293 T cells using the LipoFiter kit. Viral supernatants were collected 48 h after transfection and filtered through a 0.45 μm PES filter. The collected lentiviruses were used to infect cells. Twenty-four hours’ post-infection, the cells were cultured in medium containing 1 μg/mL puromycin (Sigma-Aldrich) for one week to select for stably infected cells.

### Quantitative RT-PCR analysis

For quantitative RT-PCR, total RNA was extracted using the TRIzol method, and 1 μg of RNA was reverse-transcribed using the ReverTra Ace qPCR RT Kit (TOYOBO) following the manufacturer’s protocol. The cDNAs were subsequently used for quantitative PCR analysis with a CFX96 Touch™ Deep Well Real-Time PCR instrument. Expression levels of all target genes were normalized to GAPDH. The primers used for RT-PCR are detailed in Table S1.

### Western blot

The collected cells were resuspended in lysis buffer containing protease inhibitors as described above. Protein samples were separated by SDS-PAGE, transferred to nitrocellulose (NC) membranes, and blocked with 8% non-fat milk for 1 h. The corresponding primary antibodies were then incubated overnight at 4 °C. The membranes were washed three times with PBST, and the secondary antibody was added and incubated at room temperature for 1 h. The membranes were then washed three times with PBST. The NC membranes were analyzed using an Odyssey infrared imaging system (LI-COR Biosciences).

### Cycloheximide chase assay

Cycloheximide (CHX) chase assays were conducted to assess the half-life of endogenous SOX2 proteins. Briefly, 2 × 10^5^ control, HOS, or 143B cells with stable AKT knockdown were seeded in 12-well plates with 100 μg/mL CHX. Cells were cultured and harvested at specified time points. Endogenous SOX2 levels were measured by western blot analysis using an anti-SOX2 antibody.

### Cell proliferation and cell viability assays

For the cell proliferation assay, cells were counted and plated in 96-well plates at a density of 1 × 10^3^ cells per well. Growth curves were generated over four days using the Cell Counting Kit 8 (CCK8; Selleck). For the cell viability assay, cells were seeded onto 96-well plates at a density of 2-3 × 10^3^ cells per well and allowed to adhere overnight. Cells were then treated with various concentrations of MK-2206, cisplatin, or their combination for 48 h. Cell viability was assessed using the CCK8 assay. For drug synergy analysis, cells were treated with single agents or their fixed-ratio combinations for 48 h. Combination Index (CI) values were calculated using CalcuSyn software. A CI value < 0.9 indicates a synergistic interaction, a CI value between 0.9 and 1.1 indicates an additive interaction, and a CI value > 1.1 indicates an antagonistic interaction.

### Colony formation assay

For the colony formation assay, cells were seeded into 12-well plates at a density of 500 cells per well and incubated for 5–7 days to evaluate their colony-forming capacity. After 5–7 days of incubation, the cells were fixed with 4% paraformaldehyde for 15 min at room temperature and stained with 0.1% crystal violet for 5 min. Following the removal of excess dye, colonies were visualized using a dissection microscope. Colonies containing more than 50 cells were counted and analyzed for colony formation activity.

### Transwell cell invasion assay

Cells (1 × 10^5^) were resuspended in serum-free DMEM medium and added to 24-well Transwell chambers (Corning) precoated with Matrigel (BD Biosciences). Serum was added to the bottom wells of the chambers to stimulate cell migration. After 24 h, cells that had migrated through the membrane were fixed with 4% paraformaldehyde for 30 min and stained with hematoxylin for 30 min. Wells were washed, and non-invading cells were removed from the upper surface of the membrane. Invading cells were counted in five random fields and expressed as a percentage of the average number of cells per field under a microscope.

### Tumor sphere formation assay

For the tumor sphere formation assay, cells were seeded in Ultra-Low Attachment 96-well plates as described previously. Briefly, cells were cultured in serum-free DMEM/F12 (Thermo Fisher Scientific, Cat. No. 11330-032) supplemented with 1% insulin (Thermo Fisher Scientific, Cat. No. 17504044), 1% B27 supplement (Thermo Fisher Scientific, Cat. No. 41400045), 100 μg/mL Penicillin/Streptomycin (Thermo Fisher Scientific, Cat. No. 15140122), 20 ng/mL human recombinant epidermal growth factor (EGF) (R&D Systems, Cat. No. 236-EG-01M), and 10 ng/mL human recombinant basic fibroblast growth factor (bFGF) (R&D Systems, Cat. No. 233-FB-025/CF) in a humidified 5% CO2 incubator at 37 °C. The serum-free medium was changed every other day. Sphere images were captured after 1 to 2 weeks. Spheres were collected, and those with a diameter greater than 40 μm were counted.

### Tissue samples

All OS tumor tissues and para-cacinoma tissues were obtained from patients surgically treated at the Department of Orthopedic Oncology and Spine Tumor Center, Shanghai Changzheng Hospital. The histologic diagnosis of OS was confirmed by two independent pathologists. Patients clinical information was collected in the Orthopedic Oncology Database cohort of Shanghai Changzheng Hospital. Our work has been carried out in accordance with the Code of Ethics of the World Medical Association (Declaration of Helsinki). This study was approved by the Ethics Committees of Shanghai Changzheng Hospital with written informed consents obtained from all patients or their legal guardians.

### Immunohistochemical staining and evaluation

Formalin-fixed paraffin-embedded breast tumor tissues were preprocessed using standard techniques, as previously described.^45^ The slides were deparaffinized with xylene, rehydrated, and antigen-retrieved in a microwave oven for 20 min. The slides were initially blocked for 1 h using MOM Blocking Reagent (Vector Labs) and then incubated overnight at 4 °C with the primary antibody, followed by a 1-hour incubation with biotinylated anti-mouse or anti-rabbit secondary antibodies. The streptavidin-HRP and DAB detection kits (Vector Labs) were used according to the manufacturer’s instructions. These slides were finally counterstained with Meyer hematoxylin. Immunohistochemical staining was scored based on the following criteria: staining intensity (I) was classified as 0 (no staining), 1 (mild staining), 2 (moderate staining), or 3 (strong staining); staining percentage (P) was categorized as 1 ( < 25%), 2 (25%–50%), 3 (51%–75%), or 4 ( > 75%). For each section, the semiquantitative score was calculated by multiplying I and P, yielding a range from 0 to 12. A score of 0–3 was considered not significant (negative), 4–8 as weakly positive, and 9–12 as strongly positive. In the analysis, low expression was defined as negative or weakly positive, while high expression was defined as strongly positive.

### Tumor xenograft transplantation experiments (PDX)

PDXs were generated from clinically annotated human osteosarcoma biopsies using previously described methods. Mice were treated drug when tumors reached ∼100 mm^3^. The group of mice which were implanted with PDXs was subsequently randomly divided into the following intraperitoneal treatments (*n* = 7 per group): Vehicle, cisplatin (DDP), MK2206, and DDP + MK2206. The MK2206 group received 20 mg/kg twice weekly, the DDP group received 4 mg/kg every 5 days, and the combination group received MK2206 (20 mg/kg twice weekly) and DDP (4 mg/kg every 5 days). Tumor measurements were performed using a Vernier caliper every other day, and mouse weights were recorded simultaneously. Tumor volumes were calculated using the formula: volume = length × width² × 0.52. On day 25, the mice were euthanized, and the tumor tissues were excised and weighed.

### Flow cytometry analysis

Tumor samples from osteosarcoma patients were collected and processed into single-cell suspensions for flow cytometric analysis. The cells were analyzed using FlowJo software (Treestar, Ashland, OR, USA).

### Statistical analysis

All experiments were conducted in triplicate unless stated otherwise. Statistical analyses and graphical representations were conducted using GraphPad Prism software (version 8.0). Statistical analyses were performed using both unpaired and paired two-sided t-tests. Significance levels were set at *P* < 0.05, *P* < 0.01, and *P* < 0.001 for all tests.

## Supplementary information

Figure S1

Figure S2

Figure S3

Figure S4

Figure S5

Figure S6

Figure S7

Supplementary Figure legend

Supplementary Table S2

Supplementary Table S1

## Data Availability

The data used or analyzed during this study are included in this article and available from the corresponding author upon reasonable request.
