# Supplementary figures and images for: Targeting AKT as a promising strategy for SOX2-positive, chemoresistant osteosarcoma
Source: Bone Res. 2025 Feb 24;13:25. doi: 10.1038/s41413-024-00395-9 (PMC11850766; doi:10.1038/s41413-024-00395-9)

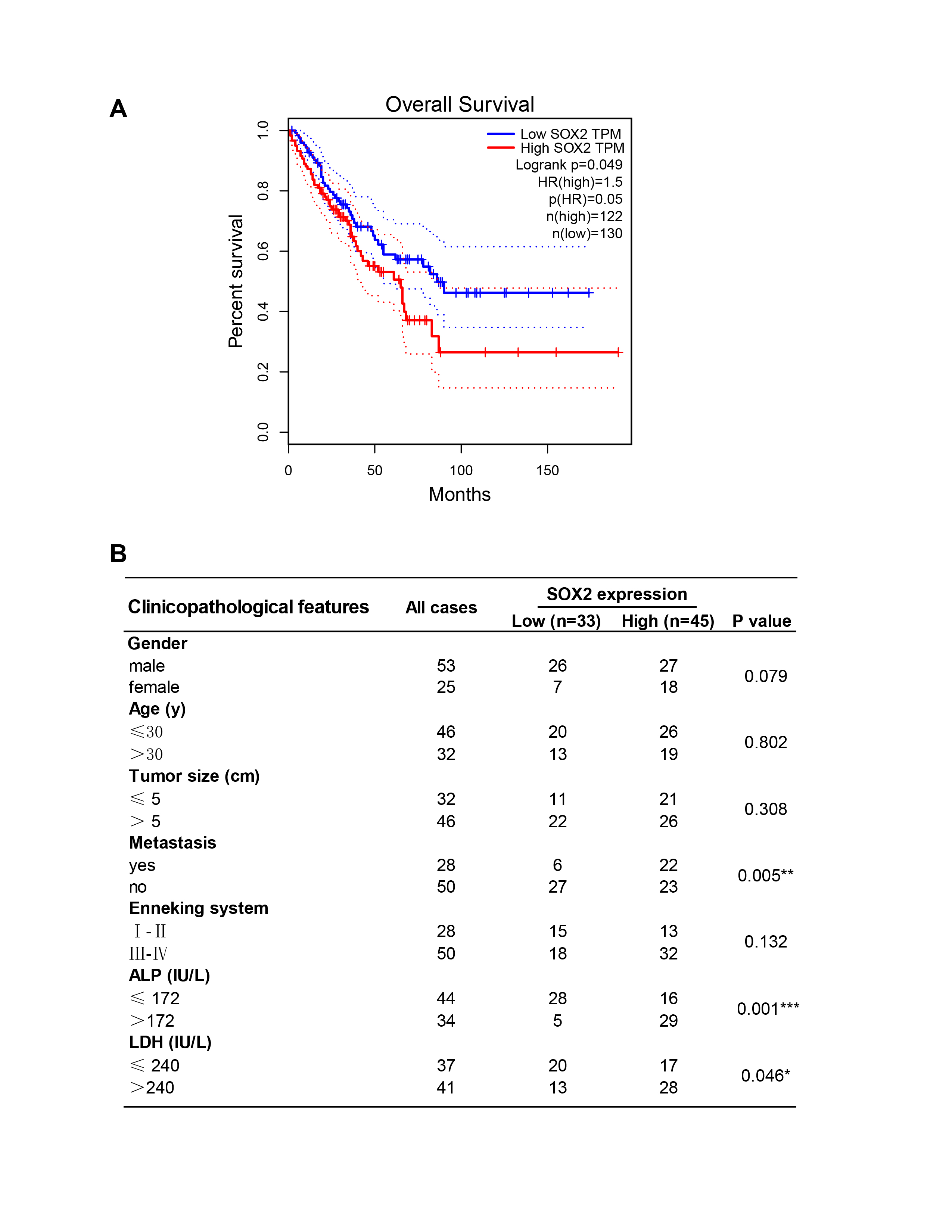

Supplement: Supplementary file 1 — Figure S1 [file 41413_2024_395_MOESM1_ESM.jpg]

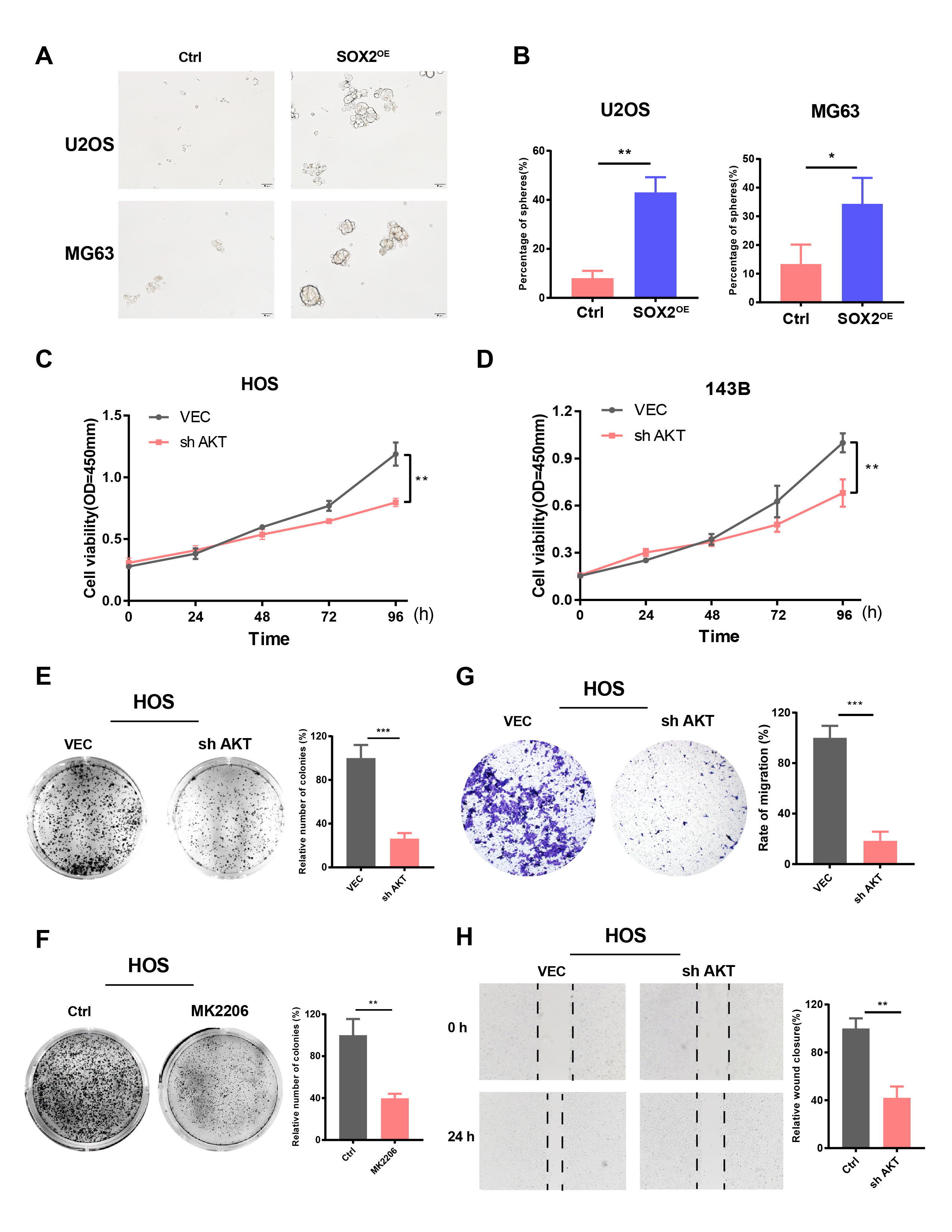

Supplement: Supplementary file 2 — Figure S2 [file 41413_2024_395_MOESM2_ESM.jpg]

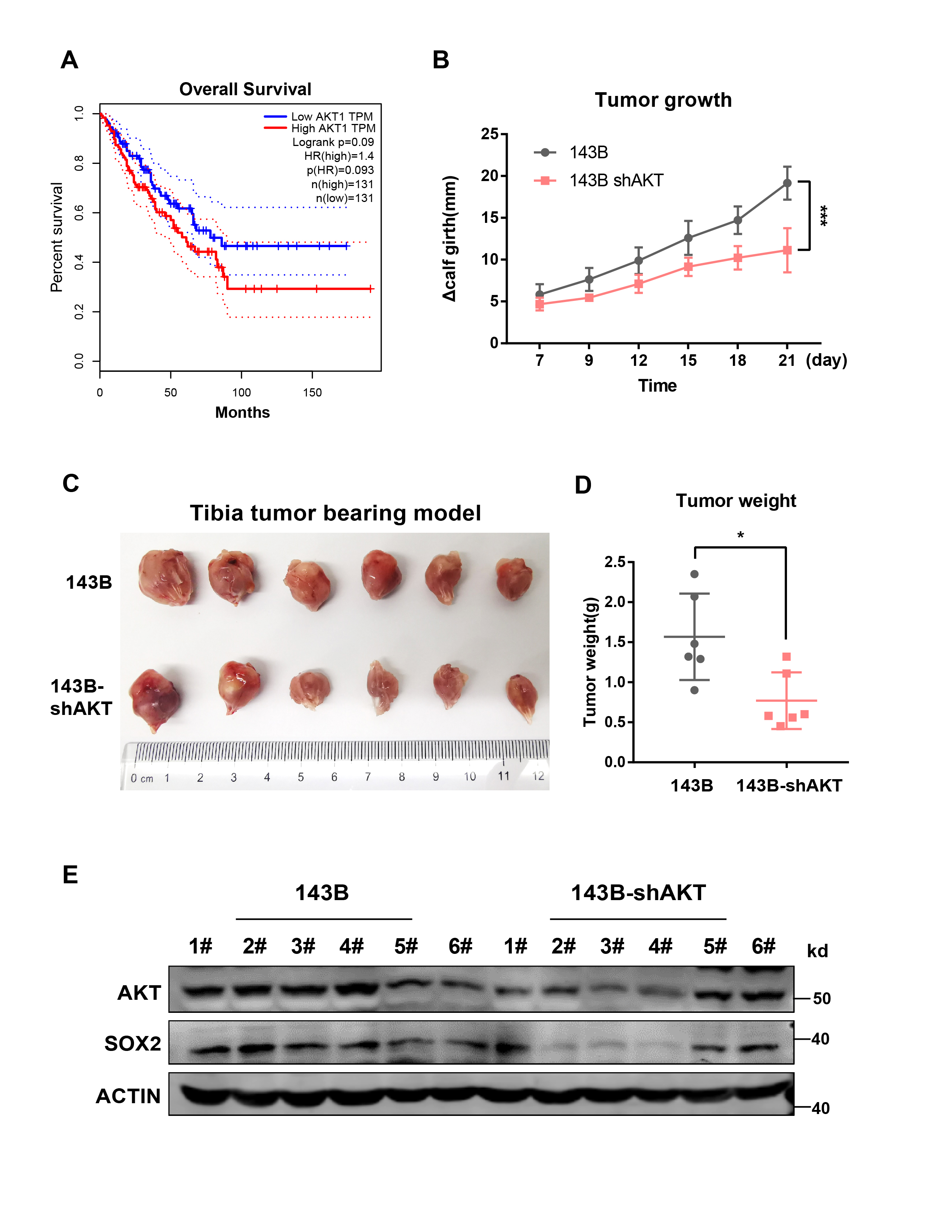

Supplement: Supplementary file 3 — Figure S3 [file 41413_2024_395_MOESM3_ESM.jpg]

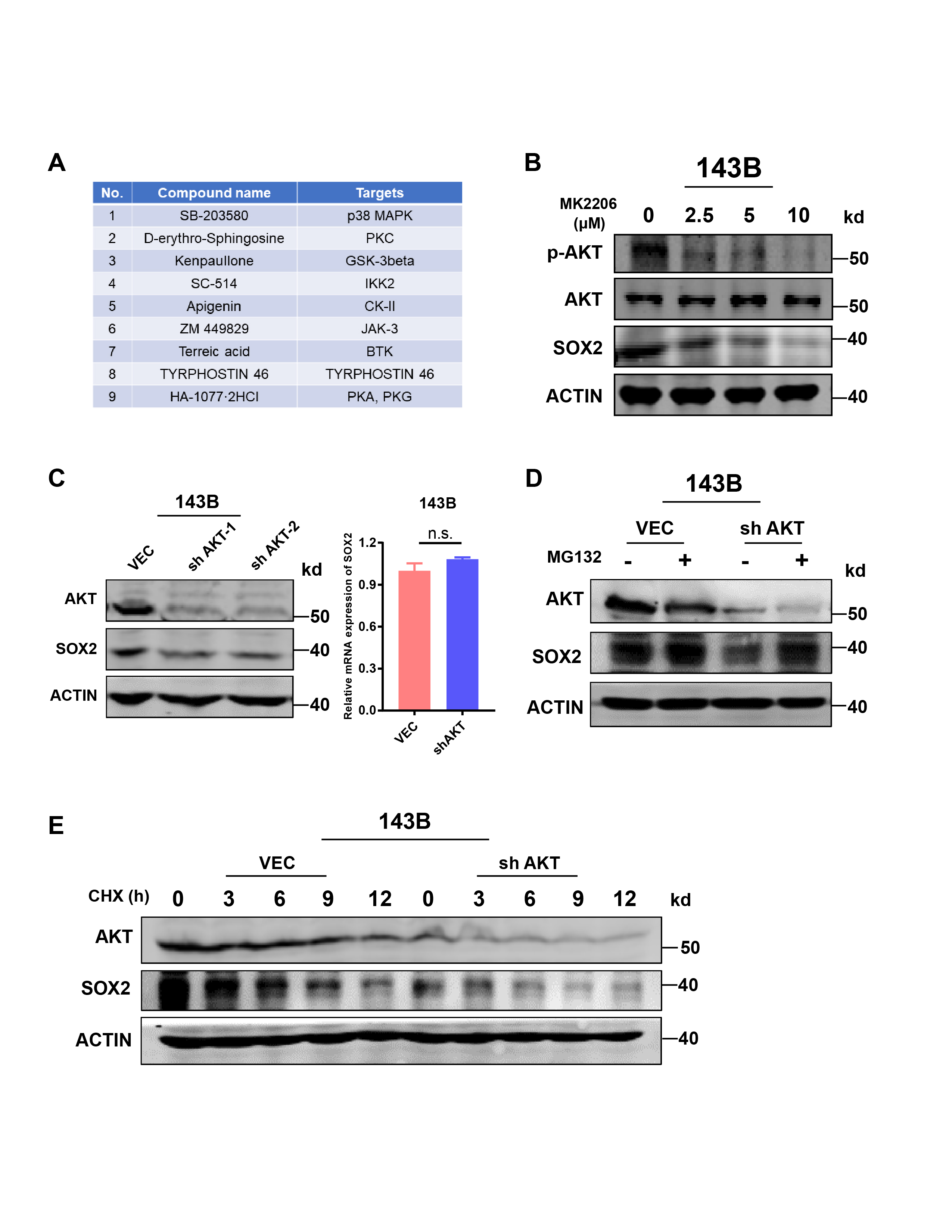

Supplement: Supplementary file 4 — Figure S4 [file 41413_2024_395_MOESM4_ESM.jpg]

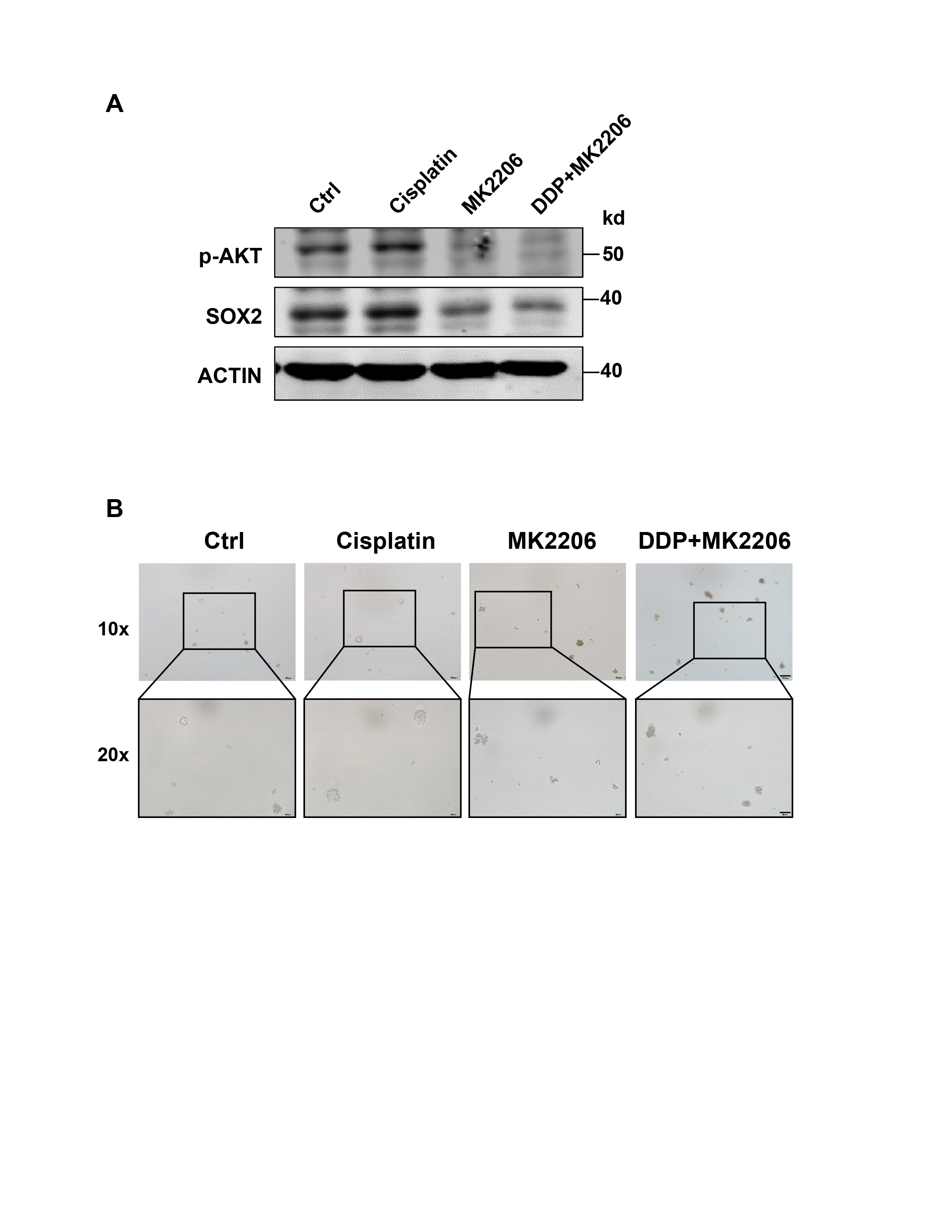

Supplement: Supplementary file 5 — Figure S5 [file 41413_2024_395_MOESM5_ESM.jpg]

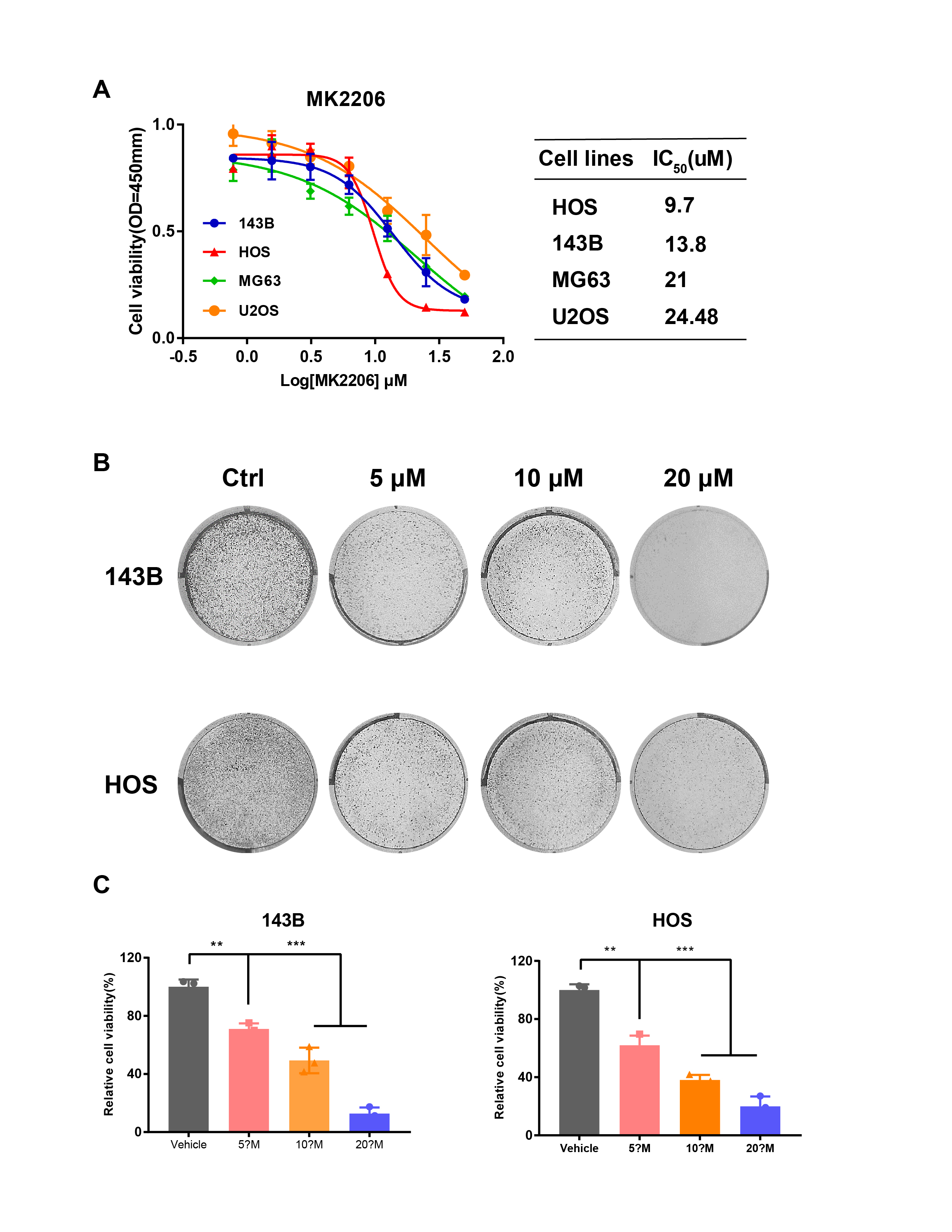

Supplement: Supplementary file 6 — Figure S6 [file 41413_2024_395_MOESM6_ESM.jpg]

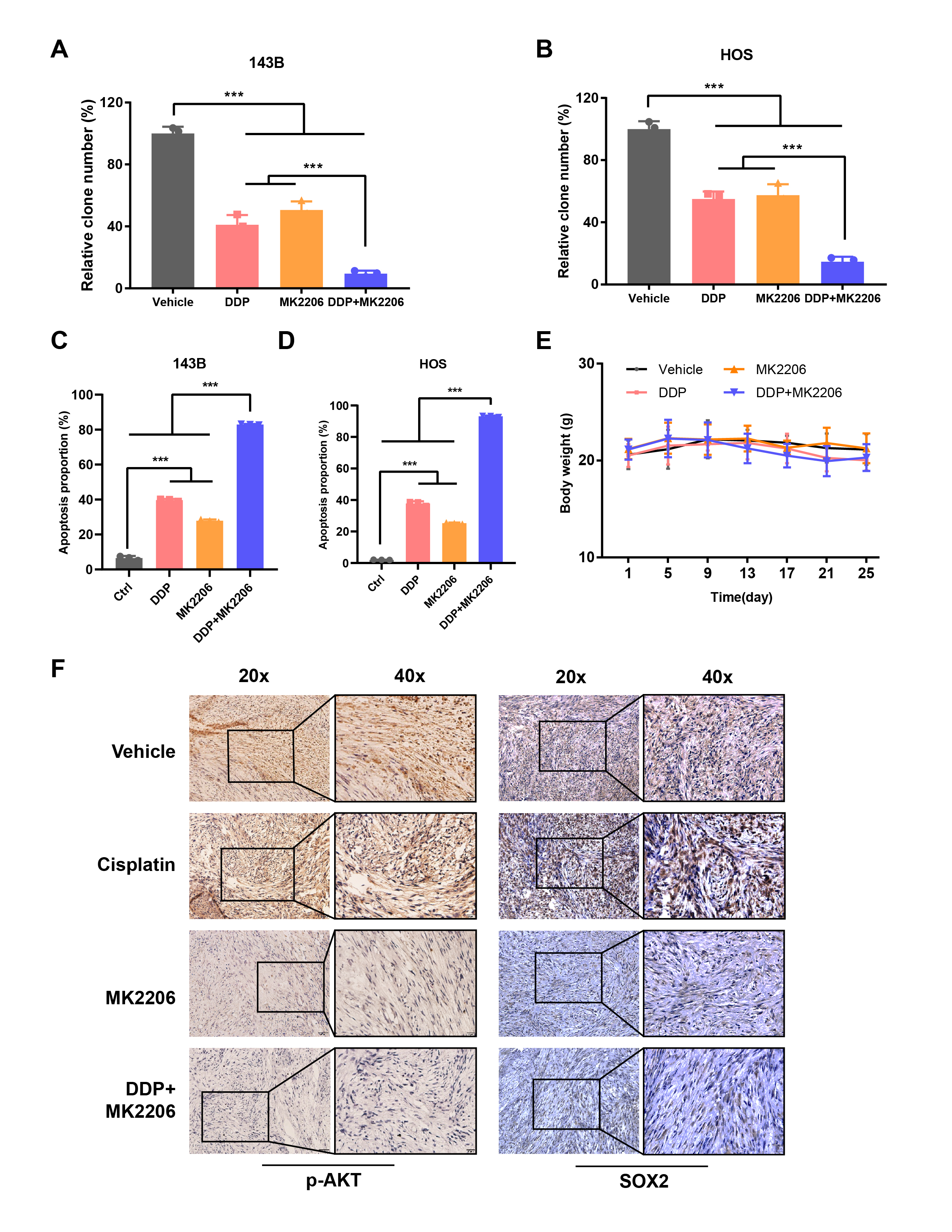

Supplement: Supplementary file 7 — Figure S7 [file 41413_2024_395_MOESM7_ESM.jpg]
