# Supplementary material for: Targeting AKT as a promising strategy for SOX2-positive, chemoresistant osteosarcoma
Source: Bone Res. 2025 Feb 24;13:25. doi: 10.1038/s41413-024-00395-9 (PMC11850766; doi:10.1038/s41413-024-00395-9)
Supplement: Supplementary file 8 — Supplementary Figure legend [file 41413_2024_395_MOESM8_ESM.docx]

**Figure S1. A.** Kaplan–Meier survival curves for sarcoma patients profiled in the GEPIA database (*http://gepia.cancer-pku.cn/*) having high (n= 122) or low (n = 130) SOX2 expression levels. High or low levels were defined based on statistically determined cutoff point that maximizes absolute value of the standardized two-sample linear rank statistic. **B.** The detailed clinicopathological features of OS patients. ^*^p < 0.05, ^**^p < 0.01, ^***^p < 0.001, p-value was measured by *chi-square* test.

**Figure S2. A.** Sarcomasphere formation comparison in control and SOX2-OE U2OS and MG63 cells. Scale bar, 100 μm (magnification, ×100). **B.** Statistical analysis of percentage of sarcomasphere Each value represents mean ± SD (n = 3); **C-F.** Cell proliferation ability detection for 143B or HOS cells with AKT knockdown or MK2206 treatment. CCK8 assay **(C, D)**, colony formation **(E, F)**. **G-H.** Cell migration ability detection for HOS ctrl cells and sh AKT cells. Transwell assay **(G)**, wound healing assay **(H)**. Each value represents mean ± SD (n=3). ^*^p < 0.05, ^**^p < 0.01, ^***^p < 0.001, p-value was measured by *t* test.

**Figure S3. A.** Kaplan–Meier survival curves for sarcoma patients profiled in the GEPIA database (*http://gepia.cancer-pku.cn/*) having high (n= 131) or low (n = 131) AKT1 expression level. High or low levels were defined based on statistically determined cutoff point that maximizes absolute value of the standardized two-sample linear rank statistic. **B.** Average values of the calf girth of tibia tumor bearing leg subtracted by the normal side leg on the same mouse during the treatment. C. Photographs of bone tumors excised from two groups mice. **D.** Statistical analysis for the tibia tumors weight. **E.** Western blot images showed that AKT and SOX2 expression in 143B and 143B-shAKT cells. Representative blots are shown from 3 independent experiments. ^*^p < 0.05, ^***^p < 0.001; p-value was measured by *t* test.

**Figure S4. A.** Kinase inhibitor and the detailed description for each inhibitor. **B.** Western blotting analysis for p-AKT, AKT and SOX2 after MK2206 treatment in gradient increase for 24 h. **C.** AKT-knockdown cells were constructed with lentiviral shRNAs against AKT and detected for WB analysis and quantitative mRNA analysis of SOX2 in 143B knockdown cells. **D.** Western blotting detection for AKT and SOX2 expression in 143B VEC and sh AKT cells with or without MG132 treatment. **E.** Intracellular protein stability assay to detect SOX2 protein stability in 143B VEC and sh AKT cells with different time period CHX treatment. Western blot images: actin is a control for protein loading. Representative blots are shown from 3 independent experiments. n.s. means no significance; p-value was measured by *t* test.

**Figure S5. A. Comparison for protein expression and sarcomasphere after cisplatin, MK2206 or combination therapy.**

**A.** P-AKT and SOX2 expression after cisplatin and MK2206 treatment by western blotting analysis. **B.** Sarcomasphere detection to evaluate the tumor stem cell formation ability for OS cells with or without MK2206 or Cisplatin treatment. Scale bar, 100 μm (magnification, ×100). **C.** UMAP for single cell analysis

**Figure S6. A**. Cell viability detection and IC_50_ quantitative value in four OS cell lines for MK2206 treatment. Relative intensity measured at OD 450 nm. **B.** Cell colony formation assay to detect the inhibition effect on 143B and HOS cells with gradient concentration MK2206. **C**. Relative cell viability of OS cells with MK2206 treatment as determined concentration by colony formation assay. ^**^p < 0.01, ^***^p < 0.001; p-value was measured by *t* test.

**Figure S7. A-B.** Relative colony number for cisplatin, MK2206 or combinative therapy treatment on 143B **(A)** and HOS **(B)** cells. **C-D.** Statistical analysis of apoptosis proportion for cisplatin, MK2206 or combined therapy treatment on 143B **(C)** and HOS **(D)** cells. **E.** The body weight changes of four therapitic group mice during the treatment. **F.** Representative immunohistochemical images of p-AKT and SOX2 expression in PDX tumors of four therapeutic groups. Scale bar, 50 μm (magnification, ×400). ^***^p < 0.001; p-value was measured by *t* test.
