# Supplementary material for: Targeting AKT as a promising strategy for SOX2-positive, chemoresistant osteosarcoma
Source: Bone Res. 2025 Feb 24;13:25. doi: 10.1038/s41413-024-00395-9 (PMC11850766; doi:10.1038/s41413-024-00395-9)
Supplement: Supplementary file 10 — Supplementary Table S1 [file 41413_2024_395_MOESM10_ESM.docx]

**Table S1. Sequences for RT-PCR primers and shRNAs**

| **RT-RNA primers**  SOX2-5’  SOX2-3’  STUB1-5’  STUB1-3’  UBR5-5’  UBR5-3’  β-Actin-5’  β-Actin-3’ | GCCGAGTGGAAACTTTTGTCG  GGCAGCGTGTACTTATCCTTCT  AGCAGGGCAATCGTCTGTTC  CAAGGCCCGGTTGGTGTAATA  GTCCATCCATTTCGTGGTTCA  CCAATTCCAATCTGTCTGGCTG  GGCACCCAGCACAATGAAGATCAA  TAGAAGCATTTGCGGTGGACGATG |
| --- | --- |
| **shRNAs**  shSOX2  shAKT  shSTUB1  shUBR5 | CTGCCGAGAATCCATGTATAT  CGCGTGACCATGAACGAGTTT  GAAGAGGAAGAAGCGAGACAT  TTGGAACAGGCTACTATTAAA |
